# Characterization of Reproductive Dormancy in Male *Drosophila melanogaster*

**DOI:** 10.3389/fphys.2016.00572

**Published:** 2016-11-24

**Authors:** Olga I. Kubrak, Lucie Kučerová, Ulrich Theopold, Sören Nylin, Dick R. Nässel

**Affiliations:** ^1^Department of Zoology, Stockholm UniversityStockholm, Sweden; ^2^Department of Molecular Biosciences, Wenner-Gren Institute, Stockholm UniversityStockholm, Sweden

**Keywords:** *Drosophila melanogaster*, diapause, reproduction, mating, metabolism

## Abstract

Insects are known to respond to seasonal and adverse environmental changes by entering dormancy, also known as diapause. In some insect species, including *Drosophila melanogaster*, dormancy occurs in the adult organism and postpones reproduction. This adult dormancy has been studied in female flies where it is characterized by arrested development of ovaries, altered nutrient stores, lowered metabolism, increased stress and immune resistance and drastically extended lifespan. Male dormancy, however, has not been investigated in *D. melanogaster*, and its physiology is poorly known in most insects. Here we show that unmated 3–6 h old male flies placed at low temperature (11°C) and short photoperiod (10 Light:14 Dark) enter a state of dormancy with arrested spermatogenesis and development of testes and male accessory glands. Over 3 weeks of diapause we see a dynamic increase in stored carbohydrates and an initial increase and then a decrease in lipids. We also note an up-regulated expression of genes involved in metabolism, stress responses and innate immunity. Interestingly, we found that male flies that entered reproductive dormancy do not attempt to mate females kept under non-diapause conditions (25°C, 12L:12D), and conversely non-diapausing males do not mate females in dormancy. In summary, our study shows that male *D. melanogaster* can enter reproductive dormancy. However, our data suggest that dormant male flies deplete stored nutrients faster than females, studied earlier, and that males take longer to recover reproductive capacity after reintroduction to non-diapause conditions.

## Introduction

Many insects respond to seasonal and other environmental changes by entering a state of dormancy, also known as diapause (Tatar and Yin, 2001; Denlinger, 2002; Denlinger and Armbruster, 2014). Depending on species diapause can occur at different stages of the life cycle, such as embryo, larva, pupa and adult, and, thus, be characterized by either arrested organismal development or, in adults, by halted reproductive maturation (Fox et al., 1959; Tauber et al., 1986; Denlinger, 2002). Larval dormancy, or dauer formation, is well characterized in the nematode worm *Caenorhabditis elegans*, where it serves to model mechanisms in lifespan extension and organismal senescence (Kenyon et al., 1993; Kimura et al., 1997; McElwee et al., 2006; Honda et al., 2008). Adult dormancy in insects is manifested as an arrest of reproduction, accompanied by decreased food ingestion, suppressed metabolism, increased stress resistance, and extended adult lifespan (Tauber et al., 1986; Tatar and Yin, 2001; Denlinger, 2002; Hahn and Denlinger, 2011; Kubrak et al., 2014; Reis et al., 2015). Hence, adult diapause is highly interesting for identifying gene networks involved in adult senescence and increased longevity (see Tatar and Yin, 2001; Kucerová et al., 2016).

Adult dormancy has been investigated in detail primarily in female insects, where ovary maturation is arrested and reproduction postponed until environmental conditions become permissive. Apart from photoperiod and temperature these conditions include food availability, humidity, effect of pheromones and cuticle proteins, population density, and presence of predators (Tauber et al., 1986; Pener, 1992; Tatar and Yin, 2001; Nylin, 2013; Denlinger and Armbruster, 2014). Depending on species and habitat the duration of adult diapause can vary from several weeks to many months or even years, and reproduction can, thus, be postponed substantially (Tauber et al., 1986; Pener, 1992; Denlinger, 2002), a life history pattern which has been particularly well studied in butterflies (Karlsson et al., 2008; McElderry, 2016). Although male diapause has been described in some species (see Pener, 1992; Tatar and Yin, 2001; Lehmann et al., 2012, 2015; Zhu et al., 2013; Ojima et al., 2015; Hand et al., 2016), surprisingly little is known about its physiology and molecular mechanisms. In fact, it is not clear to what extent mechanisms of male and female diapause are similar. Furthermore, it has been shown that not all insect species have evolved adult diapause in both males and females (Pener, 1992; Denlinger and Armbruster, 2014). Thus, there are two main mating strategies to allow for successful reproduction in insects that diapause as adults (Denlinger, 1981; Kimura, 1988; Pener, 1992). The first is that mating occurs before diapause induction and sperm are stored in the spermatheca of the females throughout dormancy, as seen in the grasshopper, *Stenocatantops splendens* (Zhu et al., 2013) and firebug, *Pyrrhocoris apterus* (Socha, 2010). In this scenario the males become redundant and die after mating, like in some wasps (Lehmann et al., 2015) and mosquitos (Ceprani et al., 2008), or spend only a short time in dormancy as in *S*. *splendens* (Zhu et al., 2013). The other strategy is that male insects also enter diapause and recover in time to join their future unfertilized mates, as seen in e.g., Colorado potato beetles, monarch butterflies, and grasshoppers (Tatar and Yin, 2001). In the butterfly *Eurema hecabe* there is an interesting combination of the two strategies, with females mating before diapause with directly developing males and then again in the spring with overwintered males (Konagaya and Watanabe, 2015).

We have recently investigated experimentally induced reproductive dormancy in female *Drosophila melanogaster* (*Canton S* strain) and established its physiological characteristics as well as genome wide effects on the transcriptome (Kubrak et al., 2014; Kucerová et al., 2016). Although the dormancy in laboratory strains of *D. melanogaster* does not qualify as a diapause in the strict sense of being induced in advance of poor environmental conditions, rather than being triggered by them (Tauber et al., 1986), we could demonstrate that *D. melanogaster* is nevertheless a genetically tractable model for dormancy and lifespan extension in other organisms. In the following we will for simplicity still use the term diapause conditions for 11°C and 10L:14D and non-diapause conditions for 25°C and 12L:12D (see also Saunders et al., 1989; Saunders, 1990; Tatar and Yin, 2001), but use the term dormancy for the state induced in *D. melanogaster*.

There are to our knowledge no published records as to whether *D. melanogaster* males also enter dormancy. Thus, we subjected newly hatched male flies to the same experimental diapausing conditions as used previously for females: 11°C and 10L:14D (Kubrak et al., 2014). We found that these conditions indeed trigger dormancy in male flies characterized by immature reproductive organs, altered energy stores and changed expression of selected genes, reflecting altered metabolism and increased stress resistance. Interestingly, dormant males are do not mate control (non-dormant) females and female flies in dormancy are not sexually attractive to males kept at 25°C and 12L:12D. Thus, reproduction is also behaviorally suppressed during dormancy.

Our findings show that in *D. melanogaster* also males can be triggered to enter dormancy within an early time window, before sexual maturation, and start reproduction after recovery when environmental conditions are permissive. Although both males and females can undergo dormancy before sexual maturation, there are sex-dependent differences in the dormancy dynamics and the expression of the dormancy phenotype. This is reflected in the fact that males recover more slowly and display a different profile of nutrient utilization and accumulation.

## Results

In female flies dormancy is diagnosed by immature previtellogenic ovaries (Saunders et al., 1989; Tatar and Yin, 2001; Kubrak et al., 2014), whereas male reproductive diapause has been characterized in several insect species using a variety of markers in different studies and therefore a clear definition is lacking. The diagnostics reported include underdeveloped testes or accessory glands, cessation of spermatogenesis, lack of sperm in seminal vesicles, suppressed sperm motility, absence of male mating behavior, and various combinations of these criteria (reviewed in Pener, 1992). To firmly establish reproductive dormancy in male *D. melanogaster* we monitored all the above-mentioned parameters.

**Figure 1:**
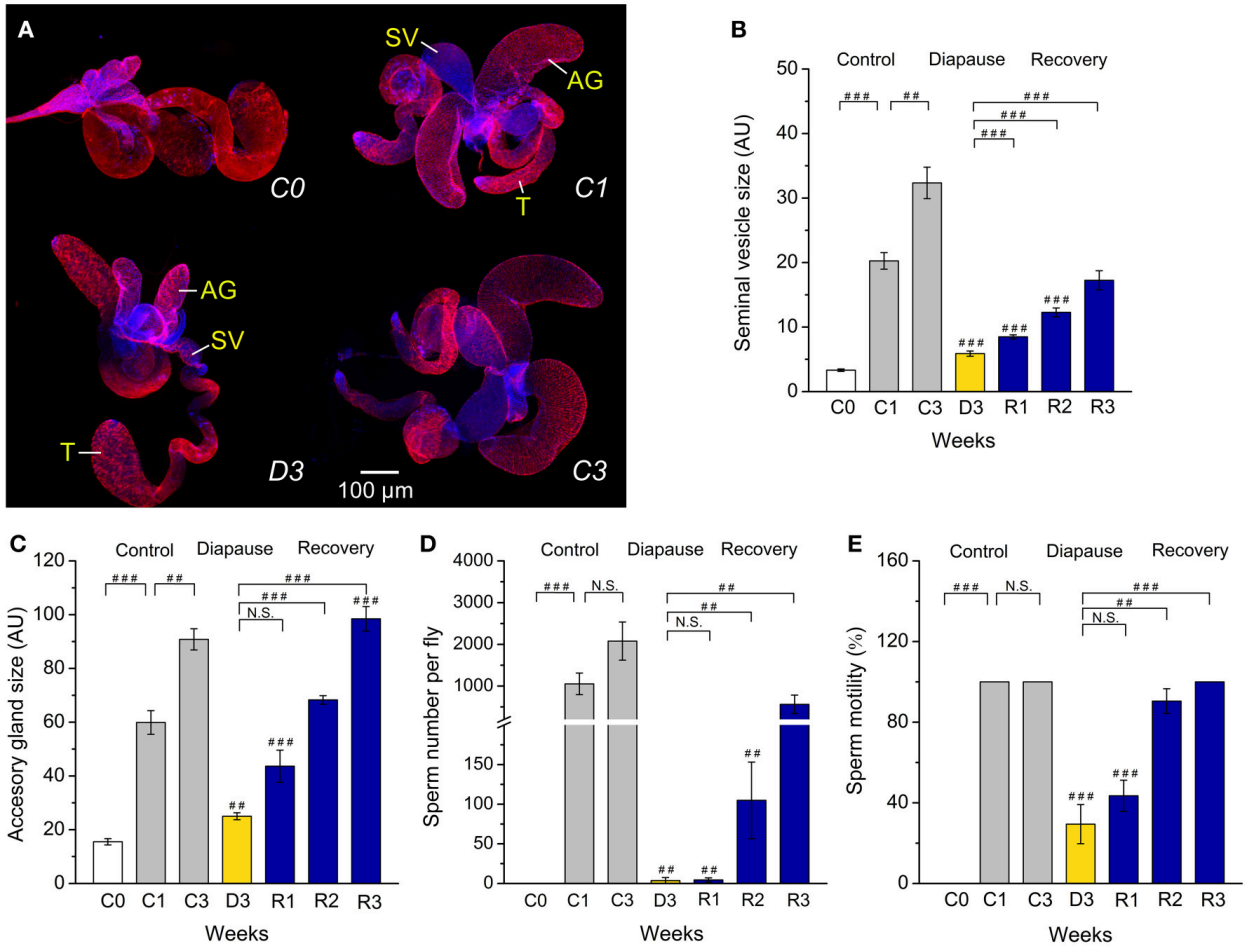
**Diapausing flies display altered reproductive organs and spermatogenesis. (A)** Unmated males (*D. melanogaster*) dormant for 3 weeks at 11°C and 10L:14D (D3) have significantly smaller seminal vesicles (SV) and accessory glands (AG) than the control flies kept for 1 or 3 weeks at 25°C and 12L:12D (C1 and C3). The size of the testis (T) does not change. The tissues were labeled with DAPI and phalloidin-rhodamine. The size of seminal vesicles **(B)** and accessory glands **(C)**, sperm number **(D)** and sperm motility **(E)** in unmated male flies, 3–6 h old (C0), kept at non-diapause conditions for 1 or 3 weeks (C1 and C3), under diapause conditions for 3 weeks (D3) and after recovery for 1–3 weeks (R1–R3). Data are presented as means ± S.E.M, *n* = 18–20 flies. Data are significantly different from the control C1 flies as indicated with ^*##*^*p* < 0.01, ^*###*^*p* < 0.001 (Kruskal–Wallis test followed by pairwise comparisons using Wilcoxon rank sum test). N.S. not significant.

In *D. melanogaster* females the readiness to enter dormancy is primarily dependent on low temperature, and only when days are longer than 16 h the flies are unable to enter dormancy (Saunders and Gilbert, 1990). A recent study in fact showed that *D. melanogaster* females readily enter dormancy at 11°C and 12L:12D conditions (Liu et al., 2016). Nevertheless, we used the same protocol as in a previous investigation of female dormancy in *D. melanogaster* where flies were kept at 11°C and 10L:14D (Kubrak et al., 2014). These conditions were also found optimal in earlier studies (Saunders et al., 1989; Saunders and Gilbert, 1990; Tatar and Yin, 2001). Thus, newly eclosed (3–6 h old), unmated male flies of the *Canton S* strain (stage designated C0) were either transferred to diapause conditions (11°C and 10L:14D) for 1 or 3 weeks (designated D1 and D3, respectively), or kept under non-diapause (control, C) conditions (25°C and 12L:12D) for 1 week (C1) or 3 weeks (C3). The non-diapausing C1 control flies were used for comparison in each assay (as in our study of female dormancy Kubrak et al., 2014). We also sampled flies that had been kept for 3 weeks under diapause conditions and then allowed to recover under non-diapause conditions for 1–3 weeks (R1–R3). The data from C0 flies are also presented as comparison since these newly eclosed animals display undeveloped reproductive organs (Figure 1) and like female flies display an immature metabolic profile (Kubrak et al., 2014).

### Dormancy affects reproductive organs and spermatogenesis

We found clear morphological differences in male reproductive organs between males, kept at 25°C for 1 or 3 weeks (C1, C3), and flies kept for 3 weeks in diapause conditions (D3) (Figure 1A). Thus, dormant males have very small seminal vesicles and male accessory glands compared to controls (C1 and C3). The immature reproductive organs of dormant males are similar to those of newly eclosed male flies (C0). Recovery from dormancy was achieved by transferring the flies back to 25°C and longer days. In flies that had recovered for 1–3 weeks (R1–R3) after 3 weeks of dormancy the seminal vesicles and accessory glands progressively increased in size, the latter eventually reached the size of 3-week-old controls (Figures 1B,C).

**Figure 2:**
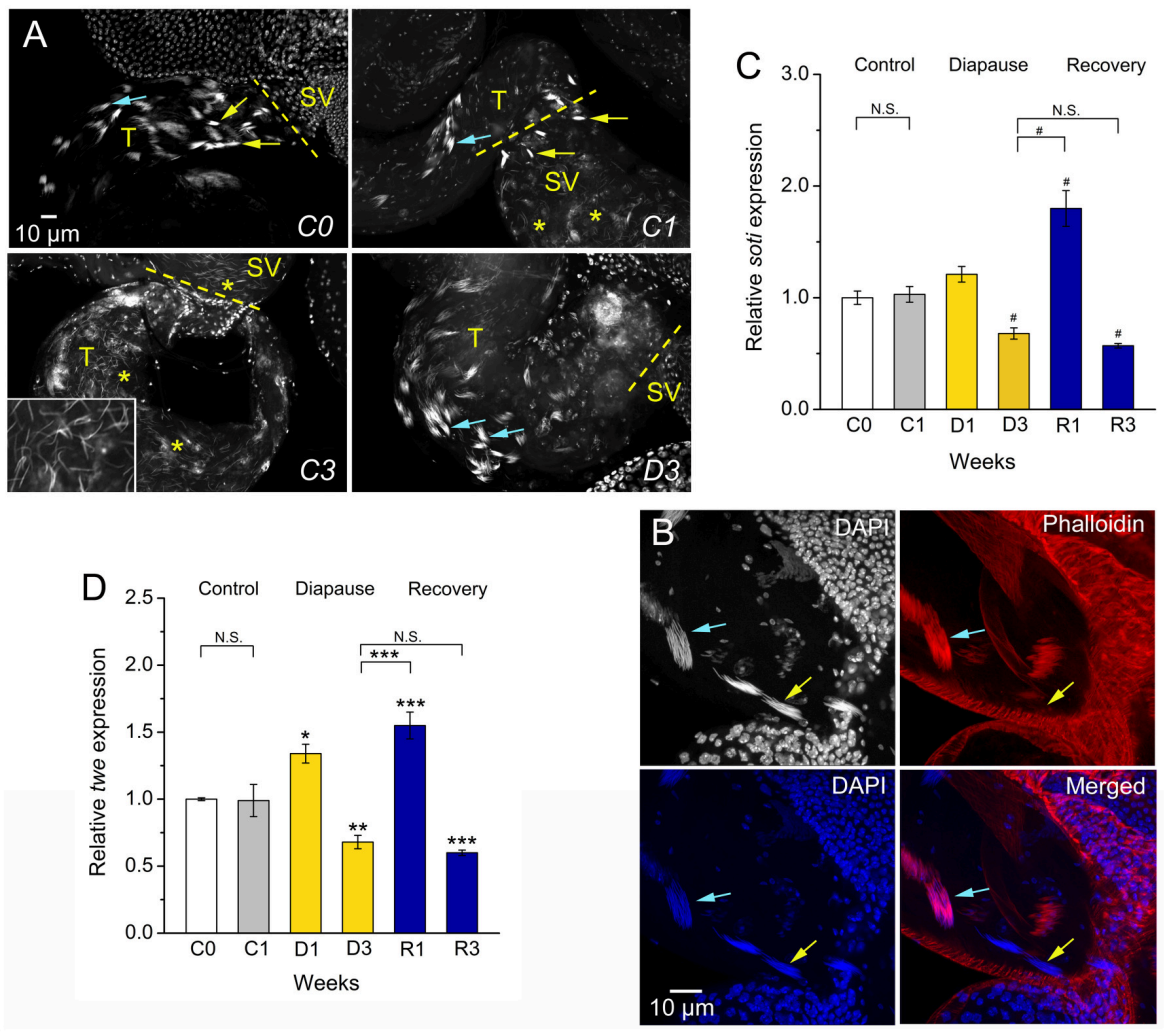
**Dormancy leads to arrested spermatogenesis at the stage of sperm individualization. (A)** Newly emerged flies (C0) and 1-week old control flies (C1) display visible early (blue arrows) and late (yellow arrows) individualization complexes in testes (T) and seminal vesicles (SV). In control (C1) flies there are many sperm nuclei (asterisks) in seminal vesicles. Seminal vesicles and testes of C3 flies are filled with sperm cells (inset shows details of sperm nuclei in testes at triple magnification). Dormant (D3) males have very few sperm in seminal vesicles, but early individualization complexes (blue arrows). **(B)** Details of early (blue arrows) and late (yellow arrows) individualization complexes in testis of control (C1) flies. Only DAPI staining is seen in late individualization complexes, whereas early ones display colocalized DAPI and phalloidin-rhodamine. **(C)**. qPCR shows that expression of *soti* (encoding Scotti, a regulator of sperm individualization) is decreased in flies dormant for 3 weeks and increases after 1 week of recovery (R1), but decreases after 3 weeks of recovery (R3). **(D)** Expression of *twe* (encoding Twine, a meiosis marker protein) is decreased in 3 week dormant flies, D3 (but not D1) and increases after 1 week of recovery (R1), but again decreases after 3 weeks (R3). Data in **(C,D)** are presented as means ± S.E.M and represent 6 replicates with 10–15 flies in each, *n* = 60–90 flies. Data are significantly different from the C1 control flies or between indicated groups with ^*^p < 0.05, ^**^p < 0.01, ^***^p < 0.001 (ANOVA followed with Tukey test) or alternatively with ^#^*p* < 0.05 (Kruskal–Wallis test followed by pairwise comparisons using Wilcoxon rank sum test). N.S. Not significant.

Since diapausing plant bug males have hypotrophied accessory glands, but still produce and accumulate significant amounts of sperm (Iarovaia and Razin, 1996), we monitored sperm number in control and diapausing, as well as recovering, *D. melanogaster* males. We found that males in dormancy have very few sperm cells (average of 5 sperm) in their seminal vesicles compared to fully filled vesicles in controls that contained between 1,000 (C1) and up to 2,500 (C3) sperm (Figure 1D). It seems that in *D. melanogaster* recovery of spermatogenesis after dormancy takes longer than recovery of oogenesis. Female flies had fully vitellogenic eggs in the ovaries after 1 week of recovery (Kubrak et al., 2014), whereas it took 3 weeks for males to completely restore sperm content after dormancy (Figure 1D). The condition of the few sperm cells produced by diapausing males was poor as determined by microscopical analysis of sperm motility (Figure 1E). A more detailed analysis of sperm production in dormant males revealed arrested spermatogenesis at the stage of sperm individualization (Bonaccorsi et al., 1990) (Figures 2A–C). Whereas, 1-week old control (C1) flies displayed clearly visible sperm individualization complexes and several thousand sperm cells in the seminal vesicles (Figure 2A), dormant males possessed very few sperm in these, but only early individualization complexes (Bonaccorsi et al., 1990) (Figure 2A). Correlated with this, the expression of *scotti* (also known as *soti*), a gene required for spermatid individualization (Fricke et al., 2014), is decreased in dormant flies (Figure 2C). Reduced meiosis during dormancy is supported by a reduction in *twine* (*twe*) mRNA, encoding the meiotic Cdc25 protein Twine (Apger-McGlaughon and Wolfner, 2013) (Figure 2D).

### Mating and fecundity is diminished in dormant flies

Male reproductive diapause results in a reversible inability of the male to inseminate receptive females (Pener, 1992), and the lack of mating appears to be part of the diapause phenotype in several insects (Swanson, 2003; Gioti et al., 2012). As expected, we found a very low number of mating attempts between dormant males and females of *D. melanogaster* (Figure S1A). We recorded no oviposition (and no progeny) after the negligible mating events between flies in dormancy (Figures S1B,C). Furthermore, we found that egg production, as well as egg fertility (scored as egg to adult viability), of flies that had recovered from dormancy is much lower than that of the control flies (Figures S1B,C). This agrees with data on the beetle *Acanthoscelides pallidipennis* where male diapause was shown to indirectly affect female reproductive performance (Chapman et al., 2003).

**Figure 3:**
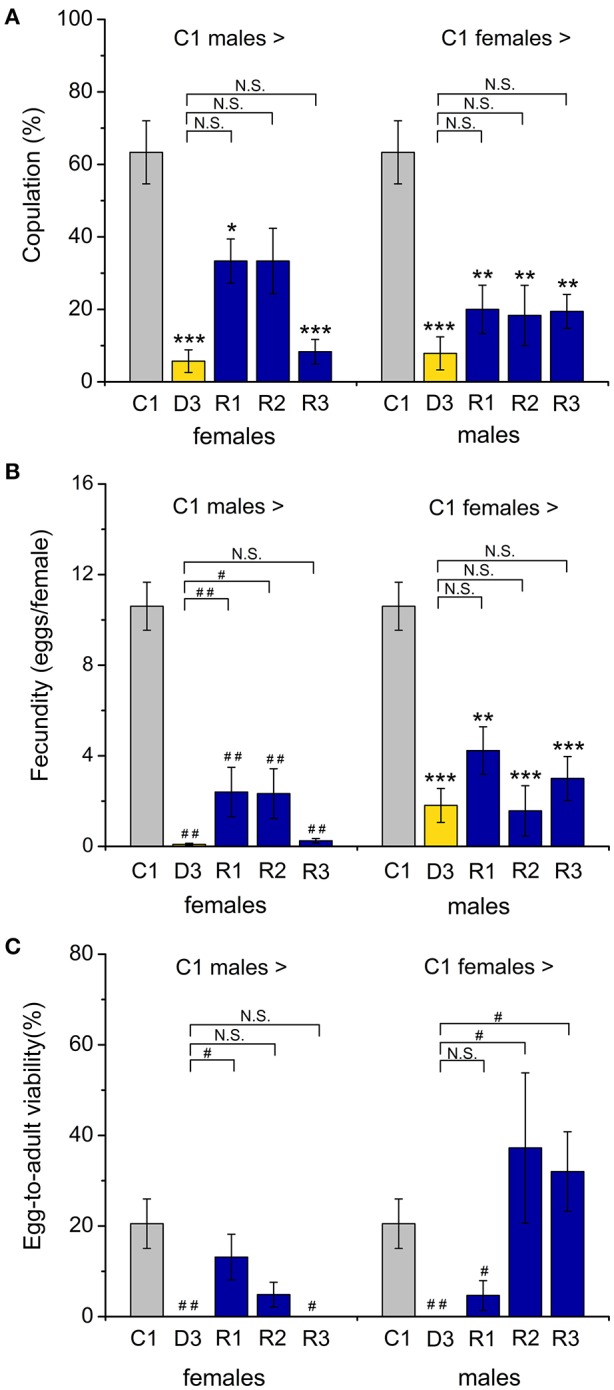
**Dormant flies display strongly reduced mating and fecundity. (A)** Number of copulation attempts of control (C1) males crossed with control (C1) diapausing (D3) or recovering (R1–R3) females. A drastic drop in copulations was observed when either male or female dormant flies were paired with controls. Recovering flies did not fully regain their rate of mating. **(B)** The number of eggs laid by a female fly after mating with dormant or recovered males (or vice versa) showed a similar reduction (compared to crosses between control partners). **(C)** The egg to adult viability is also reduced in crossings with diapausing flies, but increased for recovered flies crossed to controls, especially when the controls are females. Data are shown as mean ± S.E.M for six independent replicates with 5–7 fly couples in each replicate, *n* = 30–42 fly couples (male and female). ^*^p < 0.05, ^**^p < 0.01, ^***^p < 0.001 (ANOVA followed by Tukey test) or alternatively ^#^*p* < 0.05, ^*##*^*p* < 0.01 (Kruskal96Wallis test followed by pairwise comparisons using Wilcoxon rank sum test).

Interestingly, 1-week-old male flies kept under control conditions display strongly reduced attempts to mate with dormant females and dormant males rarely copulate with control females (Figure 3A). Also egg laying was strongly reduced when pairing a diapausing partner with a control one (Figure 3B), and the absence of progeny after crossing a dormant partner (D3) with a control one (C1) (Figure 3C) furthermore suggests that dormant partners are sterile. Males that had recovered from dormancy (R1–R3) displayed an increased tendency to mate with control females (Figure 3A), however they never reached the mating success of control males. Increased mating of the female normally results in an increase in fecundity, or fertility or both (Fan et al., 1999). In *D. melanogaster* females the fecundity of the recovered flies remained low (Figure 3B). A similar effect was seen in the post-diapausing grasshopper *S. splendens* (Zhu et al., 2013). Even after 3 weeks of recovery (R3) from diapause, when the male reproductive organs were fully mature, the number of copulation attempts was low between R3 and the control (C1) partners (Figure 3A), as well as between R3 flies of the two sexes (Figure S1A). This is also reflected in a low fecundity (egg production by females), since non-mated females lay much fewer eggs (Chapman et al., 2003). However, the egg-to-adult viability for the male progeny was fully restored after 2 weeks of recovery from dormancy (Figure 3C). This effect on males is not visible after crossing of partners that both had recovered from dormancy (compare Figure 3C and Figure S1C), probably as a result of low fecundity of the older females that had recovered (see Cirera and Aguade, 1997).

### Dormancy affects energy stores and metabolic regulation

**Figure 4:**
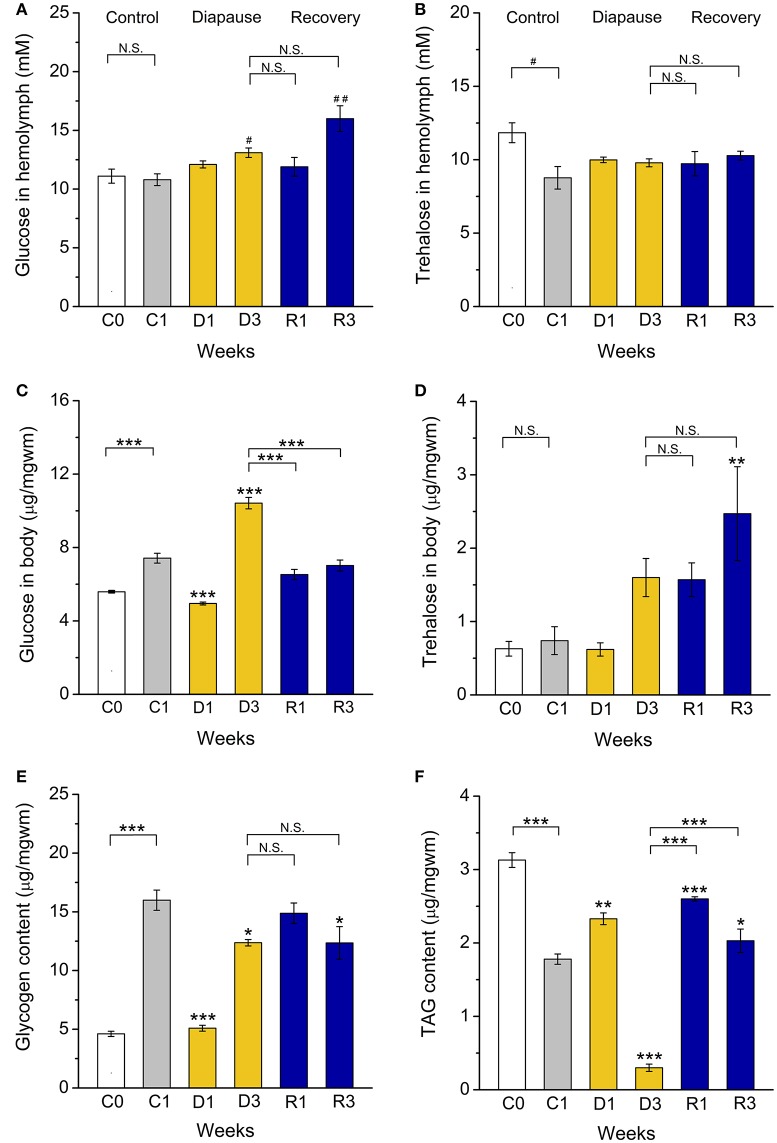
**Dormant flies display alterations in carbohydrate and lipid stores. (A)** Hemolymph glucose is increased after 3 weeks of dormancy (D3) and 3 weeks of recovery from diapause (R3). **(B)** Hemolymph trehalose is not altered during dormancy. **(C)** Whole body (stored) glucose is low after 1 week of (D1) and then increases (D3) during dormancy. **(D)** Whole body trehalose is not significantly affected by dormancy, but elevated in recovering flies (R3). **(E)** Stored glycogen is lower in flies dormant for 1 week (D1). **(F)** Triacylglycerid (TAG) content first increases (D1) and then decreases (D3) during dormancy. After 1 (R1) and 3 (R3) weeks of recovery TAG levels increased back to control levels. Data significantly different from that in the C1 control flies or between groups indicated with connectors are shown: ^*^p < 0.05, ^**^p < 0.01, ^***^p < 0.001 (ANOVA followed with Tukey test) or alternatively with ^#^*p* < 0.05, ^*##*^*p* < 0.01 (Kruskal–Wallis test followed by pairwise comparisons using Wilcoxon rank sum test). N.S. Not significant. Data represent 6 replicates with 10–15 flies in each replicate, *n* = 60–90 flies.

Extended lifespan during diapause is enabled by a strong regulation of metabolism and energy storage (Hahn and Denlinger, 2011). In addition, increased nutrient reserves are critical for post-diapause fitness (Hahn and Denlinger, 2007, 2011). Previously we found that dormant *D. melanogaster* females have lower body mass and display increased circulating and stored carbohydrates and triacylglycerides (TAG) compared to non-dormant ones (Kubrak et al., 2014). We find here that *D. melanogaster* males kept in dormancy for the same duration display a lower body weight (Figure S2) and a slight hyperglycemia, with hemolymph glucose levels higher than in C1 controls (Figure 4A). However, the circulating trehalose concentration was not affected by dormancy (Figure 4B). Whole-body levels of glucose are higher after 3 weeks of dormancy than in controls, but decreased to the control level during recovery (R1, R3) (Figure 4C). Whole body trehalose is slightly higher in males after 3 weeks of dormancy and, in contrast to glucose, did not decrease after recovery (Figure 4D). The main stored carbohydrate, glycogen, is lower at the beginning of dormancy (D1), compared to non-dormant males, then increased somewhat, but still remained at a slightly lower level than in control flies (Figure 4E). Surprisingly, the dormant male D3 flies exhausted stored TAG (Figure 4F), while females kept high levels of TAG over 12 weeks of diapause conditions (Kubrak et al., 2014). Rapid use of stored nutrients might result in a shorter dormancy in males than in females. Indeed, a shorter duration of male adult diapause was seen in several insect species (reviewed in Pener, 1992).

**Figure 5:**
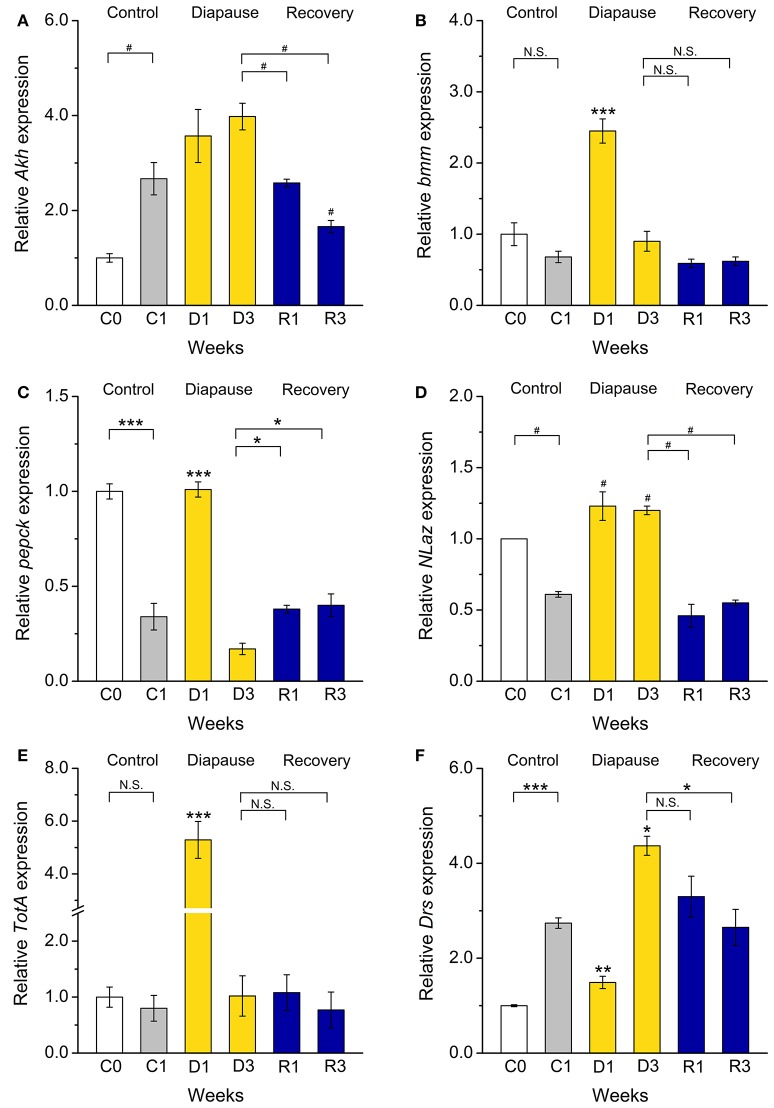
**Dormancy affects expression of genes regulating metabolism, stress responses, and innate immunity**. Using qPCR, we measured transcript levels of genes encoding **(A)** adipokinetic hormone (*Akh*), **(B,C)** the targets of IIS phosphoenolpyruvate carboxykinase (*pepck*) and Brummer lipase (*bmm*), stress-responsive genes, **(D)** *Neural Lazarillo* (*NLaz*) and **(E)** *Turandot A* (*TotA*), and **(F)** the immune gene *Drosomycin* (*Drs*). Of these *bmm, pepck, NLaz*, and *TotA* transcripts increased after 1 week of dormancy (D1) and *Drs* after 3 weeks (D3). After recovery for 1–3 weeks (R1–R3) from dormancy, transcription of all genes return to levels close to that in the control flies. Data significantly different from the C1 control flies, or between groups indicated with connectors: ^*^p < 0.05, ^**^p < 0.01, ^***^p < 0.001 (ANOVA followed with Tukey test) or alternatively ^#^*p* < 0.05 (Kruskal–Wallis test followed by pairwise comparisons using Wilcoxon rank sum test). N.S. Not significant. Data represent 6 replicates with 10–15 flies in each replicate, *n* = 60–90 flies. Results are analyzed with 2^−ΔΔCt^ method and shown as a fold of expression, normalized vs. expression in newly eclosed (C0) flies. *rp49* was used as a housekeeping gene.

In *D. melanogaster* carbohydrates and lipids are regulated by adipokinetic hormone (AKH) and insulin-like peptides (DILPs) (Rulifson et al., 2002; Bharucha et al., 2008; Owusu-Ansah and Perrimon, 2014; Padmanabha and Baker, 2014; Gáliková et al., 2015). Our investigation of female flies suggested that AKH signaling is upregulated and insulin signaling downregulated during dormancy (Kucerová et al., 2016). In the present study we did not detect a significant increase in *Akh* mRNA expression, but after recovery the levels dropped significantly (Figure 5A). This correlates with the less prominent shift in carbohydrate metabolism, and suggests a difference between males and females.

We did not monitor DILP transcript levels since we found that a better readout for insulin signaling (IIS) in diapausing flies is to measure mRNA of target genes (Kucerová et al., 2016). A downregulation of IIS in dormant *D. melanogaster* males is suggested from our data by an activation of transcription of two FOXO targets, *phosphoenolpyruvate carboxykinase* (*pepck*) and *brummer* (*bmm*) TAG lipase in flies dormant for 1 week (Wang et al., 2011) (Figures 5B,C). This was also observed in dormant *D. melanogaster* females (Kubrak et al., 2014; Kucerová et al., 2016). We also found an enhanced transcription of *bmm*, the fly homolog of adipose triglyceride lipase (ATGL) (Grönke et al., 2005), which indicates an activation of lipolysis during diapause. Finally, the increased *pepck* transcription we detected in dormant males suggests an increase of both gluconeogenesis and glyceroneogenesis (Okamura et al., 2007) and was previously recorded in diapausing larvae of the mosquito *Wyeomyia smithii* (Emerson et al., 2010) and pupae of the flesh fly *Sarcophaga crassipalpis* (Ragland et al., 2010).

### Dormancy affects stress tolerance

One of the characteristics of the diapause phenotype is enhanced stress resistance (Schmidt et al., 1993, 2005b; MacRae, 2010; Kucerová et al., 2016). This includes increased resistance to heat, cold, starvation desiccation and oxidative stress (Tatar and Yin, 2001; Schmidt et al., 2005a,b). Stress responses are reflected in activation of several conserved signaling pathways. These include the Jun-N-terminal Kinase (JNK) signaling (Biteau et al., 2011) and Janus kinase/signal transducer and activator of transcription (JAK/STAT) (Agaisse and Perrimon, 2004). The JAK/STAT signaling together with the Toll immune pathway furthermore regulate innate immune responses (Lemaitre et al., 1996; Agaisse and Perrimon, 2004; Valanne et al., 2011). We found increased expression of read-out genes from the above pathways in dormant *D. melanogaster* males (Figures 5D–F). Thus, Neural *Lazarillo* (*NLaz*) from JNK signaling (Hull-Thompson et al., 2009), *Turandot A* (*TotA*), a read-out of JAK-STAT signaling (Agaisse et al., 2003), and a target of Toll signaling, *Drosomycin* (*Drs*) (Fehlbaum et al., 1994) are up-regulated in dormant males compared to C1 controls, but are decreased back to the control values after recovery (Figures 5D–F). These results are consistent with those obtained for dormant *D. melanogaster* females (Kucerová et al., 2016) and are in accordance with a stress-resistant diapause phenotype.

## Discussion

Adult dormancy, or reproductive diapause, caused by unfavorable environmental conditions is characterized by an adaptive shift from reproduction to somatic maintenance and thereby leading to drastically extended lifespan, diminished metabolic rate, and increased stress resistance (Tauber et al., 1986; Denlinger, 2002; Emerson et al., 2009; MacRae, 2010; Hahn and Denlinger, 2011; Flatt et al., 2013). Importantly, the adult diapause therefore serves to synchronize the timing of development and reproduction with favorable seasonal conditions (Tauber et al., 1986). If mating does not occur prior to dormancy, the presence of male insects is required when the females recover. Our findings here suggest that in *D. melanogaster* both sexes can enter adult reproductive dormancy and resume reproduction when experiencing favorable conditions. We, however, find that the male reproductive system needs longer recovery time after dormancy to resume function as compared to females. This is one of a set of differences observed here between male and female dormancy in *D. melanogaster*.

Recovery of egg production, to a level of young non-dormant flies, was not observed when flies that were brought out of dormancy and mated. We found lower fecundity in post-dormancy (recovered) females, but the male reproductive capacity after 3 weeks recovery (R3) is similar to that in the ones kept for 3 weeks under control conditions (C3). It was also observed males that recovered from dormancy were less successful in mating control females. Thus, dormant males appear at disadvantage compared to non-dormant ones under our experimental conditions in the laboratory. One can therefore assume that they are only competitive when females have no other choices, as may indeed normally be the case immediately after a period of poor environmental conditions in the wild. In this context it can be noted that female discrimination against older males has been reported in several other species, and it may be beneficial for increased fertility to mate with younger males (Ottiger et al., 2000; Saudan et al., 2002). Actually a reduction in fecundity after reproductive diapause has been observed in different insect groups, including Hymenoptera (Soller et al., 1997), Lepidoptera (Kim et al., 2008), and Coleoptera (Moshitzky et al., 1996) and may be seen as a trade-off for postponed reproduction.

Although diapause has clear physiological, ecological and evolutionary advantages, it is a metabolically expensive life history strategy (Hahn and Denlinger, 2007, 2011; Emerson et al., 2009). Thus, metabolic rate is reduced during adult diapause, linked with minimal locomotion and reallocation of resources otherwise utilized for reproduction (Fox et al., 1959; Tauber et al., 1986; Hahn and Denlinger, 2007). Also, there is minimal energy intake during dormancy, since feeding is negligible in *D. melanogaster* (Kubrak et al., 2014), and reduced or absent in several other insect species (Tauber et al., 1986). This means that survival during diapause is mostly based on using stored energy. Gradual depletion of metabolic stores therefore occurs during diapause (Hahn and Denlinger, 2007). In addition, post-diapause recovery, including finalizing oogenesis and spermatogenesis, also requires using metabolic stores (Moshitzky et al., 1996; Soller et al., 1997; Jones et al., 2016; Liu et al., 2016). During dormancy males of *D. melanogaster* seem to use stored nutrients faster than females. Already after 3 weeks of dormancy there is a slightly lower level of stored glycogen and a strong depletion of stored lipids in dormant males, whereas females in the same stage of dormancy show a peak in stored nutrients (Kubrak et al., 2014). Diapause is known to alter energy storage and induce strong metabolic suppression, and even with minimal nutrient intake a maximized nutrient storage is ensured (reviewed in Hahn and Denlinger, 2007, 2011; MacRae, 2010). This seems to be the case also in other insects with adult diapause (Pener, 1992). For example, in the face fly *Musca autumnalis* and butterflies, males enter diapause more readily than females, but it lasts for a shorter time (Slaidina et al., 2009). Our study indicates that dormancy may follow different dynamics in male flies, and they need more time to recover after being placed under non-diapausing conditions.

It is known that even under favorable conditions most organisms display a trade-off between reproduction and lifespan (Humphrey et al., 2009; Hasygar and Hietakangas, 2014). In *D. melanogaster* an inverse relationship was shown experimentally between sexual activity and male lifespan (Watanabe and Sakai, 2016). Comparing dormant flies to actively reproducing ones, the latter are likely to be less stress resistant and have shorter lifespan, but develop faster and display significantly higher early-life fecundity (Schmidt et al., 2005b). In contrast, dormant flies have extended lifespan, with negligible senescence (Schmidt et al., 1993), and reproduction is postponed until after recovery.

Dormancy in *D. melanogaster* males appears to be regulated by molecular mechanisms similar to those shown for females (Kubrak et al., 2014; Kucerová et al., 2016), including downregulation of insulin and target of rapamycin (TOR) signaling and up-regulation of stress response and immune genes. However, AKH signaling appears less affected than in female flies, and the metabolic regulation appears less vigorous than in females. Overall our data suggest that male dormancy follows slightly different dynamics and might be somewhat more energy consuming than in females, although males require longer time to recover reproductive function after interrupted diapause. Nevertheless, we have shown that males of *D. melanogaster* do enter dormancy when exposed to the same conditions of low temperature and short days as used for females and it will be of great interest to determine whether the underlying mechanisms in dormancy induction (and maintenance) are the same in the two sexes. Our experiments did not address the role of photoperiod in diapause induction in male flies, and it remains to be shown whether low temperature is sufficient, or if there is a day length above which dormancy does not occur, as shown in *D. melanogaster* females (Saunders and Gilbert, 1990), or in wild strains of this species (Schmidt et al., 2005a,b). Our results demonstrate that the experimentally induced dormancy in the genetically tractable *D. melanogaster* can be used as a model not only for dormancy and lifespan in general, but also for understanding life history differences between the sexes.

## Materials and methods

### Fly husbandry and diapause induction

We used *D. melanogaster*, of the *Canton S* strain obtained from Bloomington Drosophila Stock Center (BDSC), Bloomington, IN, USA. Experimental flies were grown on a medium containing 100 g/L sucrose, 50 g/L yeast, 12 g/L agar, 3 mL/L propionic acid and 3 g/L nipagin under uncrowded conditions at 25°C and normal photoperiod (12L:12D). Reproductive dormancy in males of *D. melanogaster* was induced by the same conditions as applied before for females (Kubrak et al., 2014). Briefly, newly eclosed 3–6 h old unmated male flies (designated C0) were collected under mild CO_2_ anesthesia, put into 50 mL vials (10–15 flies per vial) with 7 mL of food medium and transferred to diapause conditions, with low temperature and short photoperiod (11°C, 10L:14D) (Saunders et al., 1989; Schmidt et al., 1993). Flies were sampled after 1 (D1) and 3 weeks (D3) of diapause. Concomitantly, newly eclosed unmated flies (C0) were kept under control conditions (25°C, 12L:12D) for 1 and 3 weeks (C1 and C3). To test the reproductive ability of flies after diapause, flies dormant for 3 weeks (D3) were transferred to control conditions (25°C and 12L:12D) and kept for 1–3 weeks to recover (R1–R3).

### Morphometric analysis and imaging of reproductive organs

The morphology of male reproductive organs was investigated in flies kept under diapause conditions for 3 weeks (D3), compared to flies kept under control conditions for up to 3 weeks (C0, C1 and C3) as well as to flies that had recovered after 3 weeks of dormancy (R1–R3). Testes and male accessory glands from 18 to 20 males for each time point were dissected in phosphate-buffered saline PBS (pH 7.2), fixed in 4% paraformaldehyde in PBS for 20 min and rinsed in PBS with 0.1% Triton twice for 10 min. Nuclei/DNA and actin filaments in samples were stained with 4′,6-Diamidino-2-phenylindole dichloride (DAPI) (Sigma-Aldrich, 1:1000 dilution) and rhodamine-phalloidin (Invitrogen, 1:1000 dilution), respectively. Samples were washed with PBS with 0.1% Triton-X three times, mounted in Fluoromount-G (SouthernBiotech) and analyzed on a Zeiss LSM 780 confocal microscope or Zeiss Axioplan 2 fluorescence microscope. The size of seminal vesicles, testes and accessory glands were analyzed from images using Image J software from NIH, Bethesda, Maryland, USA (http://rsb.info.nih.gov/ij/). Data represent areas of measured portions of reproductive organs and are shown in arbitrary units (AU), and represent mean values from measurements of 18–20 randomly selected flies for each replicate.

### Sperm count and sperm motility

Testes and accessory glands from 18 to 20 males for each time point were dissected in PBS (pH 7.2). For each experimental group three intact testes were transferred into 50 μL of saline (130 mM NaCl, 5 mM KCl, 1.5 mM CaCl_2_.2H_2_O, 2 mM Na_2_HPO_4_, 0.37 mM KH_2_PO_4_, pH 6.0), where seminal vesicles were carefully opened to release the sperm cells. After 30 min aliquots of diluted sperm solution were sampled in a Bürker CE cell counting chamber (chamber depth 0.1 mm Marca: Marienfeld, Germany) to count the number of sperm cells.

Sperm motility, the ability of sperm to move and swim forward, was scored after releasing sperm into saline. Data are shown as proportion of males with present motile sperm in testes, relative to the total number of analyzed males (40).

### Reproduction and fertility

Male mating was scored by counting copulation trials. Prior to copulation unmated males and females were kept separately in the experimental conditions on the enriched food medium (10–15 flies per a 50 mL vial with 7 mL food medium). For each mating a fly couple, 1 male and 1 female, were placed in a vial with 7 mL of food medium and the number of copulation attempts was monitored during 2 h. Dormant flies (D3) were used in mating experiments about 10 min after their removal from 11°C and 10L:14D (they were given 10 min recovery before exposure to the opposite sex). Unmated dormant (D3) or recovered (R1–R3) males were coupled with females kept at the same conditions or control females, kept for 1 week at 25°C and 12L:12D (C1). We also paired dormant females with control (C1) males. After 2 h of coupling males were removed and females left for oviposition for 24 h. After 24 h of egg laying females were discarded and number of egg was counted for each fly couple. Eggs were kept at 25°C and 12L:12D until off-spring eclosed. Copulation data are shown as a relative number (percentage) of copulation trials among total number of fly couples subjected to copulation (Partridge and Farquhar, 1981). Egg-to-adult viability is the ratio of the total number of emerged adults to the total number of laid eggs (fraction of eggs developing into adulthood) (Vijendravarma and Kawecki, 2013). Data are shown as an average of 6 independent replicates with 5–7 fly couples for each replicate (30–42 males and females).

### Carbohydrate and lipid content

We used 10–15 unmated male flies in each of 6 independent replicates from all investigated groups: control (C), dormancy (D), and recovery (R) to measure concentration of circulating (hemolymph) glucose together with stored (whole body) glucose, trehalose, and glycogen, as well as stored triacylglycerides (TAG). Hemolymph was collected following a protocol from Broughton et al. (2008) and Demontis and Perrimon (2010) including centrifugation (3000 g, 4°C, for 6 min). After hemolymph extraction the pelleted fly bodies were homogenized in PBS (pH 7.2) in ratio of 1:10 (w/v) with subsequent centrifugation (16,000 g, 4°C, 15 min) and collection of supernatants. Carbohydrate assays of hemolymph and whole body supernatants were done as described in detail in our previous study (Kubrak et al., 2014), using a glucose assay kit with glucose oxidase and peroxidase (Liquick Cor-Glucose diagnostic kit, Cormay, Poland). Concentrations of hemolymph glucose are given in mM, whereas amount of body glucose and trehalose, as well as glycogen are expressed as micrograms per milligram of wet mass of flies (μg/mgwm).

Amount of triacylglycerides (TAG) was measured by a two-step colorimetric assay using Triglyceride Reagent (Sigma-Aldrich, T2449) and Free Glycerol Reagent (Sigma-Aldrich, F6428) in accordance with (Palanker et al., 2009; Bai et al., 2012). Data are expressed as micrograms per milligram of wet mass of flies (μg/mgwm) and represent an average of 6 independent replicates with 5–7 flies in each replicate.

### Quantitative real-time PCR (qPCR)

Total RNA was isolated from 10 to 15 whole flies each from five independent biological replicates using Trizol-chloroform. Extracted RNA was further treated with DNAase (EN0521, Thermo Fisher Scientific). Quality and concentration of the RNA were determined with a NanoDrop 8000 spectrophotometer (Thermo Fisher Scientific). Reversible transcription (cDNA synthesis) was done following (Sajid et al., 2011) in 20 μL reaction mixture, containing 2 μg of total RNA, 1 μl of 10 mM dNTPs (R0192, Thermo Fisher Scientific), 0.4 μl of 100 μM random hexamer primer (SO142, Thermo Fisher Scientific), and 2 μL of M-MuLV reversible transcriptase (EP0352, Thermo Fisher Scientific) were used. The cDNA was then applied for quantitative real-time PCR (qPCR) using a StepOnePlus (Applied Biosystems) instrument and SensiFAST SYBR Hi-ROX Kit (Bioline) as recommended by the manufacturer. For each sample duplicate reactions of the total volume of 20 μl were conducted with a primer concentration of 400 nM and 4 μL of diluted 1:10 cDNA template. The mRNA levels were normalized to *rp49* levels in the same samples. Relative expression values were determined by the 2^−ΔΔCt^ method (Livak and Schmittgen, 2001). The sequences of the primers are shown in Table S1.

### Data analysis

The experimental data are presented as means ± S.E.M. Statistical analysis was performed using R Statistical Software (Foundation for Statistical Computing, Vienna, Austria) version 3.0.3. Prior to statistical treatment all data were tested for homogeneity of variances using the Fligner–Killeen test and for normal distribution by the Shapiro–Wilk normality test. Unless otherwise stated statistical analysis was performed by one-way analysis of variance (ANOVA) followed by Tukey multiple comparisons test, when data had a normal distribution, and a non-parametric Kruskal–Wallis test followed by pairwise comparisons using Wilcoxon rank sum test when data lacked normal distribution. A 95% confidence limit (P 0.05) was used throughout the study. Graphs were produced in OriginPro 7.5 software.

## Author contributions

Designed research OK, LK, UT, SN, DN; performed experiments and data analysis OK, LK; wrote manuscript OK, DN; supervised the study DN. All authors read, edited, and approved the final version of the manuscript. Obtained main funding SN.

## Funding

The study was funded by a grant from The Knut and Alice Wallenberg Foundation (KAW2012.0058) and The Swedish Research Council (VR-2012-3715) to SN. Part of the financial support was from The Swedish Research Council (VR-621-2010-5742) to DN and The Swedish Foundation for International Cooperation in Research and Higher Education (STINT, IG2011-2042) to UT. Some equipment was purchased with funding from the strategic research program EkoKlim at Faculty of Science, Stockholm University.

### Conflict of interest statement

The authors declare that the research was conducted in the absence of any commercial or financial relationships that could be construed as a potential conflict of interest.
