# Corrigendum: Characterization of Reproductive Dormancy in Male *Drosophila melanogaster*

**DOI:** 10.3389/fphys.2017.00314

**Published:** 2017-05-10

**Authors:** Olga I. Kubrak, Lucie Kučerová, Ulrich Theopold, Sören Nylin, Dick R. Nässel

**Affiliations:** ^1^Department of Zoology, Stockholm UniversityStockholm, Sweden; ^2^Department of Molecular Biosciences, Wenner-Gren Institute, Stockholm UniversityStockholm, Sweden

**Keywords:** *Drosophila melanogaster*, diapause, reproduction, mating, metabolism

It has been noted that several of the citations in this paper are incorrect. These are on pages 4–5 in the sections “Dormancy Affects Reproductive Organs and Spermatogenesis” and “Mating and Fecundity Is Diminished in Dormant Flies.” Thus, the correct citations are not in the publication list of the published paper, but are listed below. This error was caused by a problem with introduction of citations from multiple bibliography libraries.

The authors apologize for this error and state that this does not change the scientific conclusions of the article in any way.

(Fricke et al., 2014) should be replaced by (Barreau et al., 2008).

(Apger-McGlaughon and Wolner, 2013) should be replaced by (Maines and Wasserman, 1999).

(Gioti et al., 2012) should be replaced by (Krysan, 1990).

(Swanson, 2003) should be replaced by (Krysan and Higbee, 1990).

(Chapman et al., 2003) should be replaced by (Sadakiyo and Ishihara, 2012).

## Conflict of interest statement

The authors declare that the research was conducted in the absence of any commercial or financial relationships that could be construed as a potential conflict of interest.

## References

[B1] Barreau C. Benson E. Gudmannsdottir E. Newton F. White-Cooper H. (2008). Post-meiotic transcription in *Drosophila* testes. Development 135, 1897–1902. 10.1242/dev.021949 18434411

[B2] Krysan J. L. (1990). Laboratory study of mating-behavior as related to diapause in overwintering *Cacopsylla-Pyricola* (Homoptera, Psyllidae). Environ. Entomol. 19, 551–557. 10.1093/ee/19.3.551

[B3] Krysan J. L. Higbee B. S. (1990). Seasonality of mating and ovarian development in overwintering *Cacopsylla-Pyricola* (Homoptera, Psyllidae). Environ. Entomol. 19, 544–550. 10.1093/ee/19.3.544

[B4] Maines J. Z. Wasserman S. A. (1999). Post-transcriptional regulation of the meiotic Cdc25 protein Twine by the Dazl orthologue Boule. Nat. Cell Biol. 1, 171–174. 10559904 10.1038/11091

[B5] Sadakiyo S. Ishihara M. (2012). Cost of male diapause indirectly affects female reproductive performance. Entomol. Exp. Appl. 143, 42–46. 10.1111/j.1570-7458.2012.01223.x

