# Supplementary material for: Characterization of Reproductive Dormancy in Male Drosophila melanogaster
Source: Front Physiol. 2016 Nov 24;7:572. doi: 10.3389/fphys.2016.00572 (PMC5121231; doi:10.3389/fphys.2016.00572)
Supplement: Supplementary file 1 [file DataSheet1.pdf]

Supplementary Material

Characterization of reproductive diapause in male *Drosophila melanogaster*

Olga I. Kubrak, Lucie Kučerová, Ulrich Theopold, Sören Nylin, and Dick R. Nässel*

* **Correspondence:** Corresponding Author: dnassel@zoologi.su.se

Supplementary figures

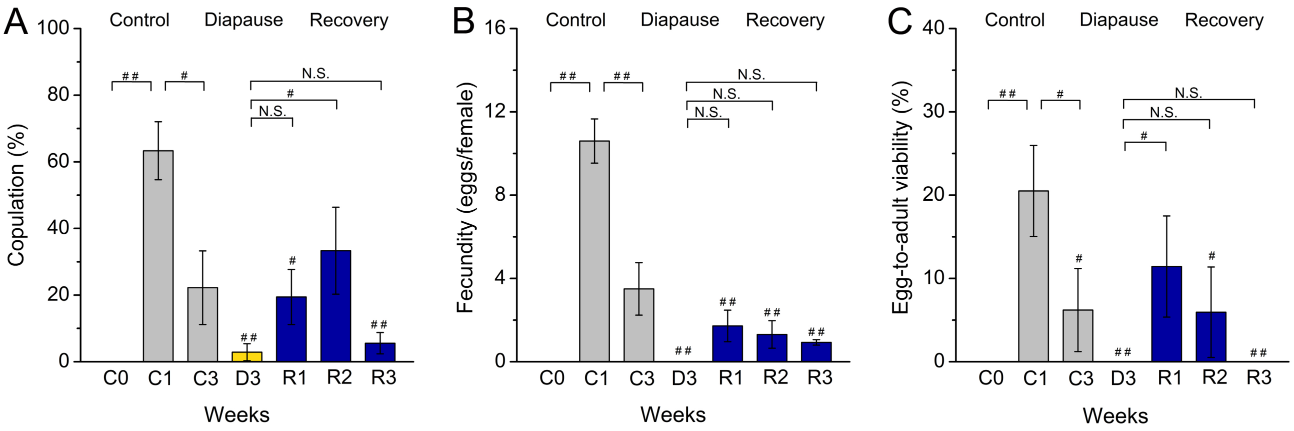

Fig. S1. Diapause conditions affect copulation, fecundity and egg to adult viability. All data are shown for crossing between identical partners (pairing virgin female - unmated male of the same condition). Newly eclosed flies (C0) of either sexe do not show any copulation behaviour and there is no oviposition or progeny. **A.** Three week diapausing flies (both, females and males) (D3) display strong reduction in copulation in comparison to the 1-week control flies, but copulation increases after recovery from diapause (R1) to the level of 3-week control (C3) flies. **B.** Fecundity after copulation of identical partners is negligible for diapausing females (D3) (crossed with diapausing males). Post-diapausing, recovered (R1-R3) females after crossing with recovered males (R1-R3) display lower fecundity than control flies (C1-C3). **C** Although female flies that recovered from diapause for 1-3 weeks (R1-R3) and then crossed with recovered male partners (R1-R3) lay fewer eggs than the controls (C1), the egg-to-adult viability of eggs produced by R1 females is similar to those of the control C1 flies. Data are shown as mean ± S.E.M , and average 6 independent replicates with 5-7 fly couples in each replicate, n = 30-42 fly couples (male and female). Data significantly different from the control C1 flies or between groups is indicated with $^{\#\#}p < 0.01$, $^{\#\#\#}p < 0.001$ (Kruskal–Wallis test followed by pairwise comparisons using Wilcoxon rank sum test). N.S. Not significant. See Fig 3 for the same parameters after crossing of C0-R3 flies with a control (C1) partner.

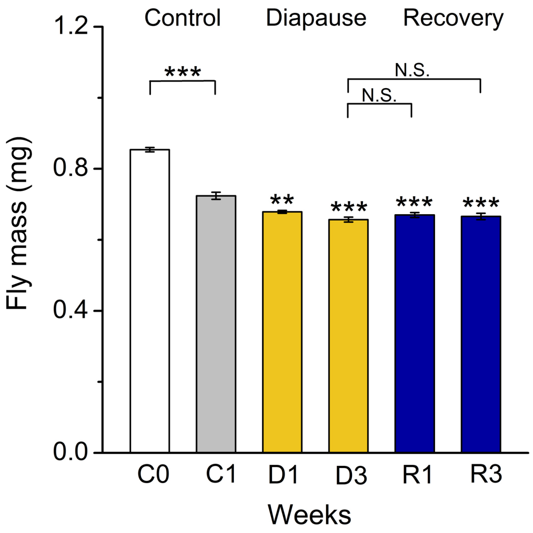

Fig. S2. The body mass of diapausing flies decreases. Fly wet mass (mg) of unmated males of *D. melanogaster,* including 3-6 h old flies (C0), and those kept in normal conditions for 1 or 3 weeks (C1 and C3), or in diapause conditions for 3 weeks (D3) and thereafter recovery for 1-3 weeks (R1-R3). Data significantly different from the controls (C1) are indicated with *p < 0.05, **p < 0.01, ***p < 0.001 (ANOVA followed with Tukey test). Data represent 6 replicates with 6-15 flies in each replicate, *n* = 36-90 flies.

Supplementary table

Table S1. Primers for qPCR (all displayed 5'–3')

Primer	Sequence	Reference
rp49 F<br>*rp49* R	ATC GGT TAC GGA TCG AAC AA<br>GAC AAT CTC CTT GCG CTT CT	Kubrak et al., 2014
Akh F<br>*Akh* R	AGA CCT CCA ACG AAA TGC TG<br>GTG CTT GCA GTC CAG AAA GAG	Hentze et al., 2015
pepck F<br>*pepck R*	TCA ATG GCG AAT CCT GCT AC<br>TCC TTC ACG TCC ACC TTA TCC	Hentze et al., 2015
bmm F<br>*bmm R*	GGT CCC TTC AGT CCC TCC TT<br>GCT TGT GAG CAT CGT CTG GT	Hentze et al., 2015
NLaz F<br>*NLaz R*	GGT GAA TGC GGC CAT CAA TC<br>AAT GGC TGC GTC GGG TAA AA	This study
TotA F<br>*TotA R*	AAT TCT TCA ACT GCT CTT ATG TGC<br>TTT GGA GTC ATC GTC CTG GG	This study
soti	TCA GCG AGG CGA GTA CTT TG<br>TCC GAG GTC CAA GAG AGG TT	This study
twe	GCA AAT AGA TCA GGA TGG CGA G<br>CTC CTG CAC GGC TGA TTT TC	This study
Drs	GCA CTC TGA TTC AAA ACC GA<br>GGT CTC GTT GTC CCA GAC	This study

Unless otherwise stated primers were designed using free online software Primer Blast, BLAST database from NIH, Bethesda, Maryland, USA (http:blast.ncbi.nlm.gov/Blast/cgi). For the rest of the primers the correspon-ding references are shown.

References to primers

Hentze, J.L., Carlsson, M.A., Kondo, S., Nässel, D.R., Rewitz, K.F. (2015) The neuropeptide allostatin A regulates metabolism and feeding decisions in *Drosophila*. Sci Rep 5,11680.

Kubrak, O.I., Kucerova, L., Theopold, U., and Nässel, D.R. (2014). The Sleeping Beauty: How Reproductive Diapause Affects Hormone Signaling, Metabolism, Immune Response and Somatic Maintenance in Drosophila melanogaster. PLoS ONE 9, e113051.
